# Supplementary material for: Genome-wide fine-mapping identifies pleiotropic and functional variants that predict many traits across global cattle populations
Source: Nat Commun. 2021 Feb 8;12:860. doi: 10.1038/s41467-021-21001-0 (PMC7870883; doi:10.1038/s41467-021-21001-0)
Supplement: Supplementary file 1 — Supplementary Information [file 41467_2021_21001_MOESM1_ESM.pdf]

Supplementary Information for:

Genome-wide fine-mapping identifies pleiotropic and functional variants that predict many traits across global cattle populations

Ruidong Xiang[1, 2], Iona M. MacLeod[2], Hans D. Daetwyler[2,3], Gerben de Jong[4], Erin O'Connor[5], Chris Schrooten[6], Amanda J. Chamberlain[2], Michael E. Goddard[1,2]

[1] *Faculty of Veterinary & Agricultural Science, The University of Melbourne, Parkville 3052, Victoria, Australia*

[2] *Agriculture Victoria, AgriBio, Centre for AgriBiosciences, Bundoora, Victoria 3083, Australia.*

[3] *School of Applied Systems Biology, La Trobe University, Bundoora, Victoria 3083, Australia*

[4] *Cooperation CRV, Arnhem, The Netherlands*

[5] *CRV Ambreed, Hamilton, New Zealand*

[6] *CRV BV, Arnhem, The Netherlands*

Files included:

Supplementary Note 1-4

Supplementary Figure 1-13

Supplementary Table 1-3

Supplementary Note 1. The across-sex design of generating two sets of local gEBV for markers prioritized in bulls and cows.

The across-sex design was first, to reduce the bias that can arise if discovery, training and prediction is undertaken in the same population and second, to leverage the power from two independent populations. As shown in Supplementary Figure 1, the average genomic relationship between the bull and cow populations was around 0. We illustrate this across-sex analysis step-by-step using the following figure (Supplementary Figure 2). Three major steps were described: step 1: variant prioritization (discovery), step 2: BayesRC [1] and [2] BayesR modelling (training) and step 3: local gEBV calculation (prediction).

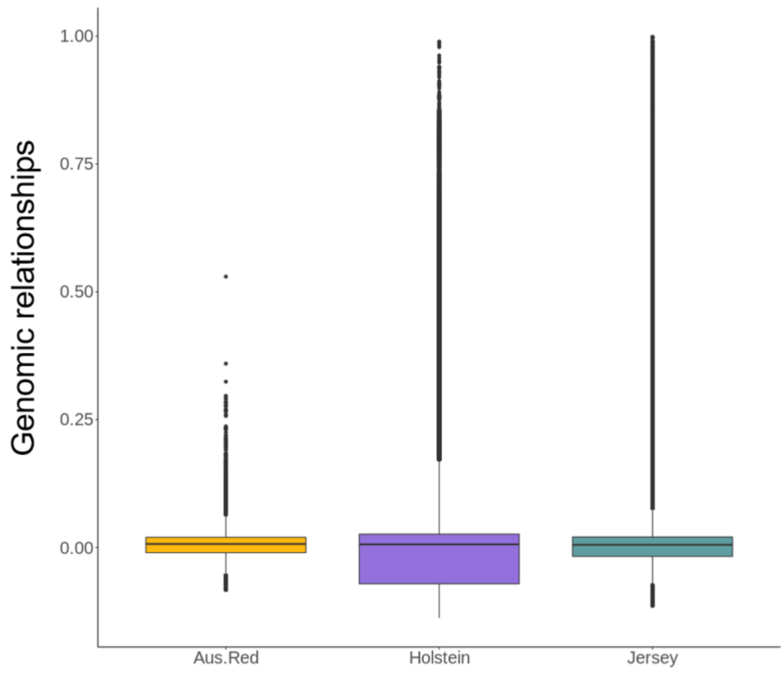

Supplementary Figure 1. The distribution of the genomic relationships between bull and cow populations for breeds used in the discovery analysis: Australian Red (Aus.Red, n=125 ♂ × 424 ♀), Holstein (n=9,739 ♂ × 22,899 ♀) and Jersey (2,059 ♂ × 6,174 ♀). The genomic relationships (across-sex off-diagonal elements) were extracted from the genomic relationship matrix made by GCTA [3] using sequence variants with MAF >0.001 in 44,000+ bulls and cows. For each box, the minimum is the lowest point, the maximum is the highest point, whiskers are maxima 1.5 times of interquartile range, the bottom bound, middle line and top bound of the box are the 25th percentile, median and the 75th percentile, respectively.

The process of discovery of variants in bulls, training in cows and bulls, and predicting into cows were visualized in Supplementary Figure 2a. Firstly, 165K variants were prioritized (discovered) in bulls. Then, these variants discovered in bulls were trained in cows (blue text in orange boxes) with BayesRC (step 2.1). BayesRC training in cows produced marker effects which will be used to calculate local gEBV into the cow population (step 3.1). Another product from the BayesRC training in cows (step 2.1) was the mixing proportions of the markers. For a BayesRC run, there may be 150,000 markers with 0 effects, 14,960 markers each contributing 0.0001 of the additive genetic variance, 33 each contributing to 0.001 and 7 each contributing to 0.01 (Methods and equation 6 in the main text). This mixing proportion ([150,000, 14,960, 33, 7]) will be used as the starting α value (instead of [1, 1, 1, 1,]) for the Dirichlet prior [2] for the BayesR run in bulls (step 2.2) which will significantly influence each BayesR iteration. Such BayesR training in the bull population will produce marker effects which will be used to predict local gEBV in the cows, again (step 3.2). Then, two sets of local gEBV estimated in the cow population using the same markers discovered from the bull population, were produced.

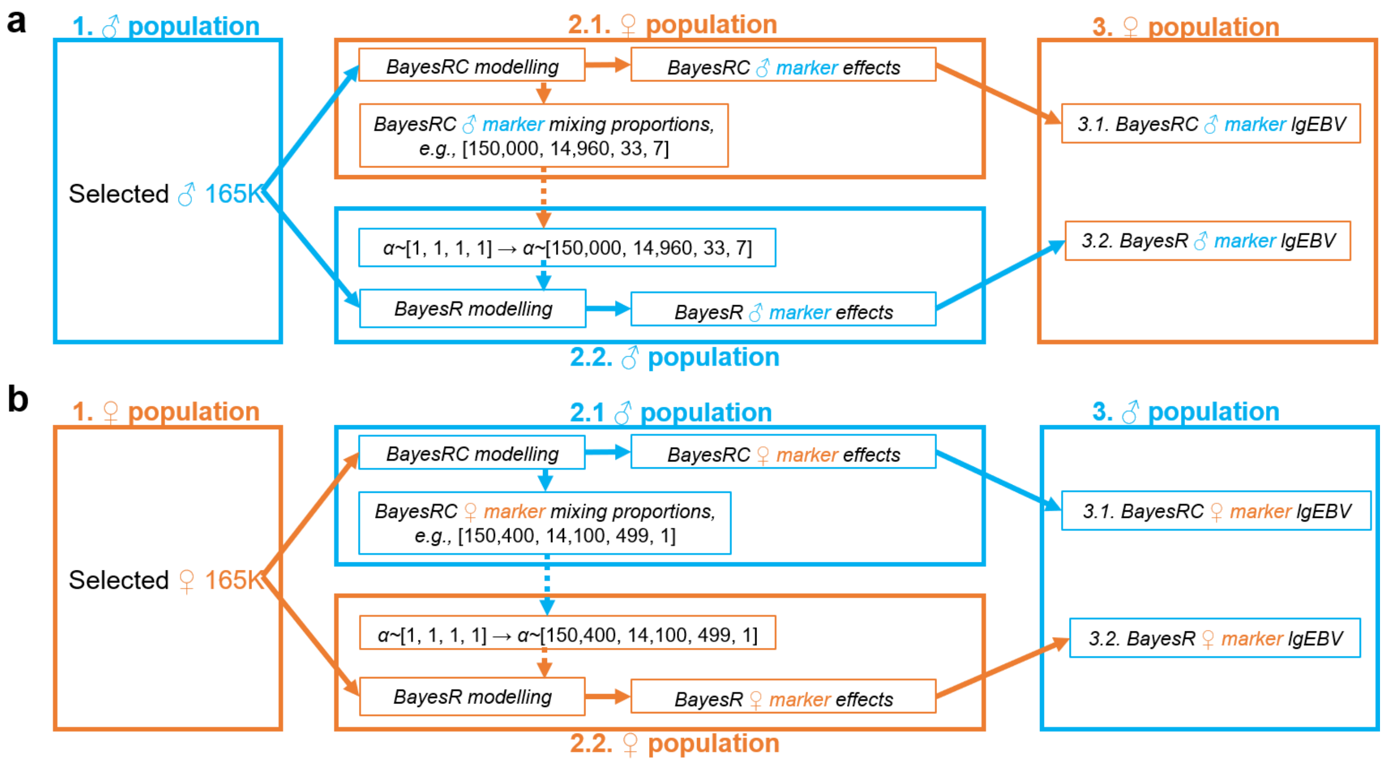

Supplementary Figure 2. The flowchart of the cross-sex design for generating two sets of local gEBV for markers prioritized (discovered) from bulls and cows.

The process of discovery of variants in cows, training in bulls and cows, and predicting into bulls were visualized in Supplementary Figure 2b. Firstly, 165K variants were prioritized (discovered) in cows (step 1). Then, these variants discovered from cows were trained in bulls (orange text in blue boxes) with BayesRC (step 2.1) and cow markers trained in bulls were used to predict bull local gEBV (step 3.1). BayesRC training in bulls produced marker effects which will be used to calculate local gEBV into the bull population (step 3.1). Another product from the BayesRC training in bulls (step 2.1) was the mixing proportions of the markers. For a BayesRC run, there may be 150,400 markers with 0 effects, 14,100 markers each contributing 0.0001 of the additive genetic variance, 499 each contributing to 0.001 and 1 each contributing to 0.01. This mixing proportion ([150,400, 14,100, 499, 1]) will be used as the starting α value (instead of [1, 1, 1, 1,]) for the Dirichlet prior [2] for the BayesR run in cows (step 2.2) and will significantly influence each BayesR iteration. This BayesR training in the cow population will produce marker effects which will be used to predict local gEBV in the bull population, again (step 3.2). Then, two sets of local gEBV estimated in the bull population using the same markers discovered from the cow population, were produced.

Supplementary Note 2. The programmatic calculation for the weighted correlation per each segment.

For the calculation of the weighted correlation for each segment, an asymmetric variance-covariance matrix obtained from BayesRC [1] and BayesR [2] (Figure 3a) was used. For each variance-covariance matrix, only the diagonal elements with positive values and their associated rows and columns in the matrix were considered for the calculation. This was to eliminate unreliable estimates of the local gEBV values which did not agree between the BayesRC and BayesR mapping for the same trait.

Because there were negative off-diagonal element (covariance) values in the matrices for many segments, simply summing the off-diagonal elements up would reduce the value of the numerator of equation 2, leading to an underestimated value of the weighted correlation which will inflate the estimation of the n(QTL) for some segments. On the other hand, the sum of the absolute value of the off-diagonal elements would increase the value of the numerator which will shrink the estimation of the n(QTL) for some segments. To properly account for the impact of negative off-diagonal values on the sum, we used an 'approximate absolute value' which was implemented as a programmatic sign-flipping process for the asymmetric matrix for each segment:

1. Determine the negative diagonal elements; if there are negative diagonal elements, discard the columns and rows associated with these negative diagonal elements

2. Determine the firstly-appeared 'trait' of which the row and column contained the largest number of negative off-diagonal elements and count the total number of negative off-diagonal elements

3. Flip the sign of all the off-diagonal elements of this trait and count the total number of negative off-diagonal elements

4. Compare the total number of negative off-diagonal elements between before and after the sign-flipping; if the total number of negative off-diagonal elements reduced after the sign-flipping, then, repeat step 1-3; if the total number of negative off-diagonal elements remained the same or became large after the sign-

flipping, then, stop, revert back to the state before the sign-flipping and keep the matrix before the sign-flipping for calculating the weighted correlation

An example of a 10 × 10 matrix was given to demonstrate this workflow in the following:

tr01	4.7E-05	-5.6E-04	-5.7E-04	8.9E-04	-1.3E-05	-3.6E-05	-9.6E-07	-3.0E-06	-2.2E-06	2.4E-06
tr02	-3.7E-05	4.9E-04	5.0E-04	-7.9E-04	9.7E-06	2.8E-05	1.0E-06	2.0E-06	1.4E-06	-1.6E-06
tr03	-9.2E-05	1.2E-03	1.3E-03	-1.9E-03	2.7E-05	7.4E-05	2.3E-06	5.7E-06	5.9E-06	-4.4E-06
tr04	3.2E-04	-4.5E-03	-4.6E-03	7.4E-03	-8.4E-05	-2.5E-04	-9.8E-06	-1.6E-05	-1.4E-05	1.3E-05
tr05	-8.5E-06	9.5E-05	9.8E-05	-1.6E-04	2.5E-06	6.2E-06	2.0E-07	4.3E-07	5.4E-08	-4.4E-07
tr06	-1.7E-05	2.0E-04	2.0E-04	-3.1E-04	4.7E-06	1.2E-05	3.9E-07	9.8E-07	4.2E-07	-8.5E-07
tr07	4.6E-06	-5.7E-05	-5.9E-05	9.3E-05	-1.2E-06	-3.5E-06	-1.2E-07	-3.0E-07	-2.1E-07	2.3E-07
tr08	-1.9E-05	2.2E-04	2.2E-04	-3.5E-04	5.3E-06	1.4E-05	4.2E-07	1.1E-06	5.2E-07	-9.8E-07
tr09	-3.1E-06	2.2E-05	2.5E-05	-4.3E-05	1.2E-06	1.7E-06	7.7E-08	4.5E-08	-4.3E-07	-1.6E-07
tr10	-4.5E-06	4.9E-05	4.9E-05	-7.6E-05	1.2E-06	3.3E-06	6.8E-08	3.1E-07	2.1E-07	-2.5E-07
	tr01	tr02	tr03	tr04	tr05	tr06	tr07	tr08	tr09	tr10

Supplementary Figure 3. An example of 10 × 10 the asymmetric variance (diagonal elements, bold border) and covariance (off-diagonal elements) matrix to start the weighted correlation analysis. Cells of the negative off-diagonal elements were colored in grey whereas cells of the positive off-diagonal elements were colored in yellow. The negative diagonal elements for trait 7, 9 and 10 were in red text. In this example, the 7th row and column, 9th row and column and 10th row and column will be excluded for the weighted correlation analysis.

tr01	4.7E-05	-5.6E-04	-5.7E-04	8.9E-04	-1.3E-05	-3.6E-05	-3.0E-06
tr02	-3.7E-05	4.9E-04	5.0E-04	-7.9E-04	9.7E-06	2.8E-05	2.0E-06
tr03	-9.2E-05	1.2E-03	1.3E-03	-1.9E-03	2.7E-05	7.4E-05	5.7E-06
tr04	3.2E-04	-4.5E-03	-4.6E-03	7.4E-03	-8.4E-05	-2.5E-04	-1.6E-05
tr05	-8.5E-06	9.5E-05	9.8E-05	-1.6E-04	2.5E-06	6.2E-06	4.3E-07
tr06	-1.7E-05	2.0E-04	2.0E-04	-3.1E-04	4.7E-06	1.2E-05	9.8E-07
tr08	-1.9E-05	2.2E-04	2.2E-04	-3.5E-04	5.3E-06	1.4E-05	1.1E-06
	tr01	tr02	tr03	tr04	tr05	tr06	tr08
No. '-'	10	4	4	10	4	4	4
No. '-' total	20						

Supplementary Figure 4. After the rows and columns for trait 7, 9 and 10 were excluded as described above, the firstly-appeared trait that had the largest number of negative off-diagonal values was trait 1 (red text). The row and column associated with trait 1 were labelled in red border and the sign of the off-diagonal elements in those cells with the red border will be flipped (× -1). The count of the number of the negative off-diagonal elements for each trait (No. '-') and the total number of the negative off-diagonal elements in the matrix (No. '-' total) were indicated in the figure. Cells of the negative off-diagonal elements were colored in grey whereas cells of the positive off-diagonal elements were colored in yellow.

tr01	4.7E-05	5.6E-04	5.7E-04	-8.9E-04	1.3E-05	3.6E-05	3.0E-06
tr02	3.7E-05	4.9E-04	5.0E-04	-7.9E-04	9.7E-06	2.8E-05	2.0E-06
tr03	9.2E-05	1.2E-03	1.3E-03	-1.9E-03	2.7E-05	7.4E-05	5.7E-06
tr04	-3.2E-04	-4.5E-03	-4.6E-03	7.4E-03	-8.4E-05	-2.5E-04	-1.6E-05
tr05	8.5E-06	9.5E-05	9.8E-05	-1.6E-04	2.5E-06	6.2E-06	4.3E-07
tr06	1.7E-05	2.0E-04	2.0E-04	-3.1E-04	4.7E-06	1.2E-05	9.8E-07
tr08	1.9E-05	2.2E-04	2.2E-04	-3.5E-04	5.3E-06	1.4E-05	1.1E-06
	tr01	tr02	tr03	tr04	tr05	tr06	tr08
No. '-'	2	2	2	12	2	2	2
No. '-' total	12						

Supplementary Figure 5. After the sign-flipping for the off-diagonal elements for trait 1 as described above, the total number of the negative off-diagonal elements in the matrix reduced (20 before VS 12 after). Therefore, the process continues to determine the firstly-appeared trait that had the largest number of negative off-diagonal values. In this example, this trait is trait 4 (red text). Therefore, the row and column associated with trait 4 were labelled in red border and the sign of the off-diagonal elements in those cells with the red border will be flipped (× -1). The count of the number of the negative off-diagonal elements for each trait (No. '-') and the total number of the negative off-diagonal elements in the matrix (No. '-' total) were indicated in the figure. Cells of the negative off-diagonal elements were colored in grey whereas cells of the positive off-diagonal elements were colored in yellow.

tr01	4.7E-05	5.6E-04	5.7E-04	8.9E-04	1.3E-05	3.6E-05	3.0E-06
tr02	3.7E-05	4.9E-04	5.0E-04	7.9E-04	9.7E-06	2.8E-05	2.0E-06
tr03	9.2E-05	1.2E-03	1.3E-03	1.9E-03	2.7E-05	7.4E-05	5.7E-06
tr04	3.2E-04	4.5E-03	4.6E-03	7.4E-03	8.4E-05	2.5E-04	1.6E-05
tr05	8.5E-06	9.5E-05	9.8E-05	1.6E-04	2.5E-06	6.2E-06	4.3E-07
tr06	1.7E-05	2.0E-04	2.0E-04	3.1E-04	4.7E-06	1.2E-05	9.8E-07
tr08	1.9E-05	2.2E-04	2.2E-04	3.5E-04	5.3E-06	1.4E-05	1.1E-06
	tr01	tr02	tr03	tr04	tr05	tr06	tr08
No. '-'	0	0	0	0	0	0	0
No. '-' total	0						

Supplementary Figure 6. After the sign-flipping for the off-diagonal elements for trait 4 as described above, the total number of the negative off-diagonal elements in the matrix reduced (12 before VS 0 after). Therefore, the process continues to determine the firstly-appeared trait that had the largest number of negative off-diagonal values. Although no negative off-diagonal elements were left, as the design of the calculation dictates, trait 1 will still be the firstly-appeared trait that had the largest number (0) of negative

off-diagonal values. Therefore, the sign of the off-diagonal elements in those cells with the red border associated with trait 1 will be flipped (× -1).

tr01	4.7E-05	-5.6E-04	-5.7E-04	-8.9E-04	-1.3E-05	-3.6E-05	-3.0E-06
tr02	-3.7E-05	4.9E-04	5.0E-04	7.9E-04	9.7E-06	2.8E-05	2.0E-06
tr03	-9.2E-05	1.2E-03	1.3E-03	1.9E-03	2.7E-05	7.4E-05	5.7E-06
tr04	-3.2E-04	4.5E-03	4.6E-03	7.4E-03	8.4E-05	2.5E-04	1.6E-05
tr05	-8.5E-06	9.5E-05	9.8E-05	1.6E-04	2.5E-06	6.2E-06	4.3E-07
tr06	-1.7E-05	2.0E-04	2.0E-04	3.1E-04	4.7E-06	1.2E-05	9.8E-07
tr08	-1.9E-05	2.2E-04	2.2E-04	3.5E-04	5.3E-06	1.4E-05	1.1E-06
	tr01	tr02	tr03	tr04	tr05	tr06	tr08
No. '-'	12	2	2	2	2	2	2
No. '-' total	12						

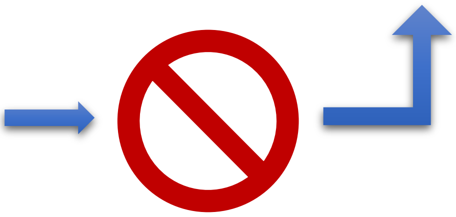

Supplementary Figure 7. After the sign-flipping for the off-diagonal elements for trait 1 as described above, the total number of the negative off-diagonal elements in the matrix increased (0 before VS 12 after). Therefore, the sigh-flipping will stop, revert to the previous step and save the matrix before this sign-flip for the weighted correlation with equation 2 as described in the main text.

Supplementary Note 3. Comparison of genomic prediction accuracy using different variant selection methods.

To further illustrate the advantage of different variant selection methods, we trained 8 sets of variants with data of 6 traits of 28.1k Australian cows and predicted into additional 14.1k Australian cows. Those 28.1k Australian cows were the same cows used as the training population in the main text to predict the EBV of New Zealand cow traits (Figure 5a) which consisted of 24.4k Holsteins, 2.5k Jerseys and 1.2k Australian Reds (described in Methods in the main text). The additional 14.1k validation Australian cows contained 10.6k Holsteins, 2.1k Jerseys and 1.4k Australian Reds. The 28.1k training and 14.1k validation cows had no overlap with those 44,000 animals used in the main text to prioritise the variants from the XT-50K panel. The phenotypes for 6 traits of 28.1k training cows and 14.1k validation cows were obtained from the official May 2020 DataGene (https://datagene.com.au/) national dairy cattle evaluations, and they included: protein yield (Prot, $N_{reference}$= 21,270, $N_{validation}$= 13,430), fat yield (Fat, $N_{reference}$= 21,270, $N_{validation}$= 13,430), milk yield (Milk, $N_{reference}$= 21,270, $N_{validation}$= 13,430), protein percentage (ProtP, $N_{reference}$= 21,270, $N_{validation}$= 13,430), fat percentage (FatP, $N_{reference}$= 21,270, $N_{validation}$= 13,430) and somatic cell count (Scc, $N_{reference}$= 20,823, $N_{validation}$= 13052).

The following 8 lists of variants were tested for their accuracy in prediction of gEBV for the 6 traits using above described design: 1) FAETHTop10k: top 10k variants based on their Functional-And-Evolutionary Trait Heritability (FAETH) ranking [4]; 2) FAETHTop80k: top 80k variants based on their ranking; 3) GWASTop10k: top 10k variants ranked based on their p-value of 34-trait GWAS meta-analysis in Australian bulls and cows [5]; 4) GWASTop80k: top 80k variants ranked based on their p-value of 34-trait GWAS meta-analysis in Australian bulls and cows; 5) FAETH.GWAS.Cluster+BayesRCTop.PP10k: top 10k variants ranked based on their summed posterior probability (PP) across 34 traits from BayesRC runs in bulls and cows, using the 165k SNPs after the clustering of top 10% variants based on their FAETH and GWAS ranking (reflecting top variants from the step 3 in the main text); 6) FAETH.GWAS.Cluster+BayesRCTop.PP80k: top 80k variants ranked based on their summed PP across 34 traits from BayesRC runs in bulls and cows; 7) FAETH.GWAS.Cluster+BayesRC.lgEBVTop80k: top 80k

variants corresponded to the selection of 80k variants as shown in Figure 1 in the main text, which were ranked based on their summed correlation squared with the variance of local gEBV from the BayesRC runs (equation 3 and step 4 in the main text) and 8) XT-50K: the final selection of 46k variants from the designed XT-50K panel. The rationale behind selecting top 10k variants based on their ranking of FAETH, GWAS and BayesRC was to test if large effect variants identified by those analysis can increase genomic prediction accuracy. The rationale behind selecting top 80k variants based on the ranking their FAETH, GWAS and BayesRC was to match the number of SNPs used in the top 80k variants we selected based on the variance of local gEBV. A proportion of our final 80K set had low beadchip array design scores and could not therefore be added to the XT-50K panel.

The training of above variant lists used single-trait BayesR [2] with the model of $\mathbf{y} = \mathbf{Xb} + \mathbf{Wv} + \mathbf{e}$ where **y** was the vector of each decorrelated trait; **X** was the design matrix allocating phenotypes to fixed effects; **b** was the vector of the fixed effect of breed; **W** was the design matrix of marker genotypes; centred and standardised to have a unit variance; **v** was the vector of variant effects, distributed as a mixture of the four distributions (described above); **e** = vector of residual errors. and the prediction of gEBV used $\widehat{y_v} = W_{1:n}\hat{v}_{1:n}$, where $\widehat{y_v}$ was the gEBV, $W_{1:n}$ was the design matrix of marker genotypes for 1 to n and $\hat{v}_{1:n}$ was the variant effects from the training dataset. The accuracy of prediction was estimated as the Pearson correlation *r* between gEBV and the individual phenotype within each breed of the validation cows.

Across 6 traits and 3 breeds (18 scenarios), 17 out of 18 times the 80k variants selected based on the lgEBV from BayesRC (‘FAETH.GWAS.Cluster+ BayesRC.lgEBVTop80k’) or the XT-50K variants topped the genomic prediction accuracy amongst other variant selections (Supplementary Figure 8). The 1 exception was that BayesRC PP ranking (‘FAETH.GWAS.Cluster+BayesRC.PPTop80k’) topped the prediction accuracy of milk yield in Jersey. Apart from the set of BayesRC lgEBV top80k and the XT-50K variants, the top 80k variants selected based on FAETH ranking (‘FAETHTop80k’) and based on BayesRC PP ranking (‘FAETH.GWAS.Cluster+BayesRCTop.PP80k’) showed competitive performances in prediction accuracies across traits and breeds. Across all scenarios, GWAS based on top variant selection had the worst prediction accuracies, compared to other variant selection methods (Supplementary Figure 8).

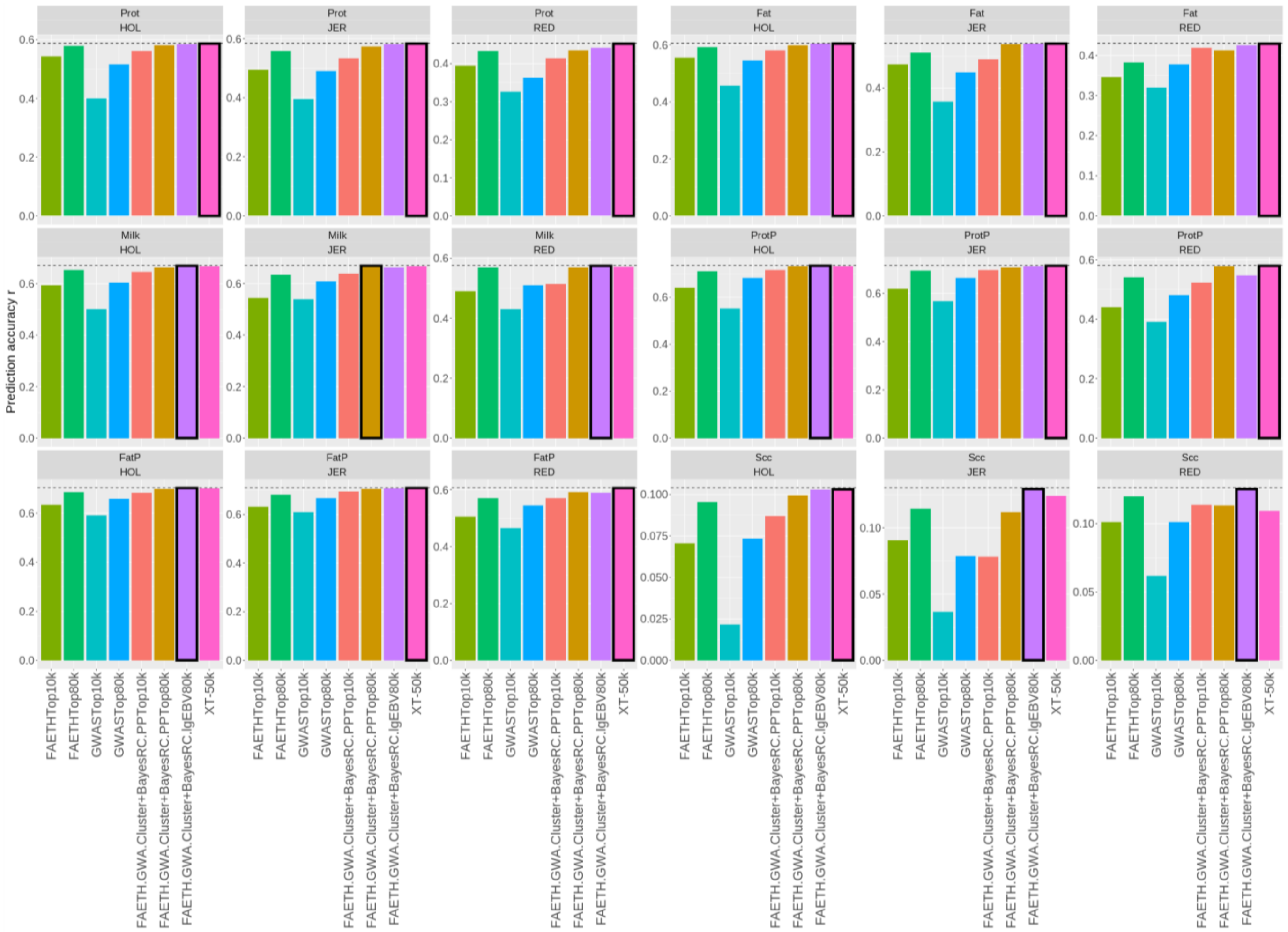

Supplementary Figure 8. Genomic prediction accuracy across 6 traits and 3 breeds using 8 different genotype sets where different methods were applied to select variants. The bar with a black border indicates the variant set that showed the highest prediction accuracy (*r*) of any given variant set within each breed by trait validation. The horizontal black dashed line indicates the prediction accuracy for the best variant set. The 8 lists of selected variants are (from left to right): FAETHTop10k and FAETHTop80k: top 10k or 80k variants, based on their Functional-And-Evolutionary Trait Heritability (FAETH) ranking; GWASTop10k and GWASTop80k: top 10k or 80k variants, ranked based on their p-value of 34-trait GWAS; FAETH.GWAS.Cluster+BayesRC.PPTop10k and FAETH.GWAS.Cluster+BayesRC.PPTop80k: top 10k and 80k variants, ranked based on their summed posterior probability across 34 traits from BayesRC, using the 165k SNPs after the clustering of top 10% variants based on their FAETH and GWAS ranking; FAETH.GWAS.Cluster+BayesRC.lgEBVTop80k: top 80k variants ranked based on their summed

correlation squared with the variance of local gEBV from BayesRC; and XT-50K: the final selection of 46k variants from the designed XT-50K panel.

Supplementary Note 4. Comparison of genomic prediction accuracy of different existing bovine SNP chip panels.

Our study aims at prioritising a set of variants that can be put on routinely used genotyping panels that usually have up to 50k markers. Ideally, we could whole-genome sequence, or use high-density panels to genotype every animal in national breeding programs. For example, the HD panel that contains up to 800,000 SNPs is a useful genotyping tool. Also, there is a recently developed GGP-F250 panel which contains up to 250,000 SNPs, many of which are potentially functional SNPs [6], largely based on *in silico* functional prediction [7]. However, whole-genome or high-density genotyping of large populations is very expensive. Therefore, a panel with a small marker number such as 50k, enriched with potentially causal variants that provide a similar genomic prediction power to high-density panels, would be optimal for large-scale genotyping. Such an informative panel can make the genotyping of animals very cost-effective to many farmers.

To compare the prediction accuracy of our XT-50K with other existing panels, including the standard-50K, HD800K and GGP-F250 [6], we performed genomic prediction analysis using SNPs from these four panels. Up to 50k SNPs from the XT-50K and standard-50K, up to 600k SNPs from the HD [8] and up to 250k SNPs from GGP-F250 were trained using BayesR and used to predict gEBV of the 6 traits in 3 breeds as described in Note S3.

Across 6 traits and 3 breeds (18 scenarios), 7 out of 18 times the XT-50K markers topped the genomic prediction accuracy and another 6 out of 18 times the HD markers topped the genomic prediction accuracy (Supplementary Figure 9). 5 out 18 times the GGP-F250 markers topped the genomic prediction accuracy (Supplementary Figure 9). These results show that the predictive power of the XT-50K, with much smaller number of markers, is at least as good as denser panels such as GGP-F250 and HD. The standard 50K panel had the worst prediction accuracy across all scenarios.

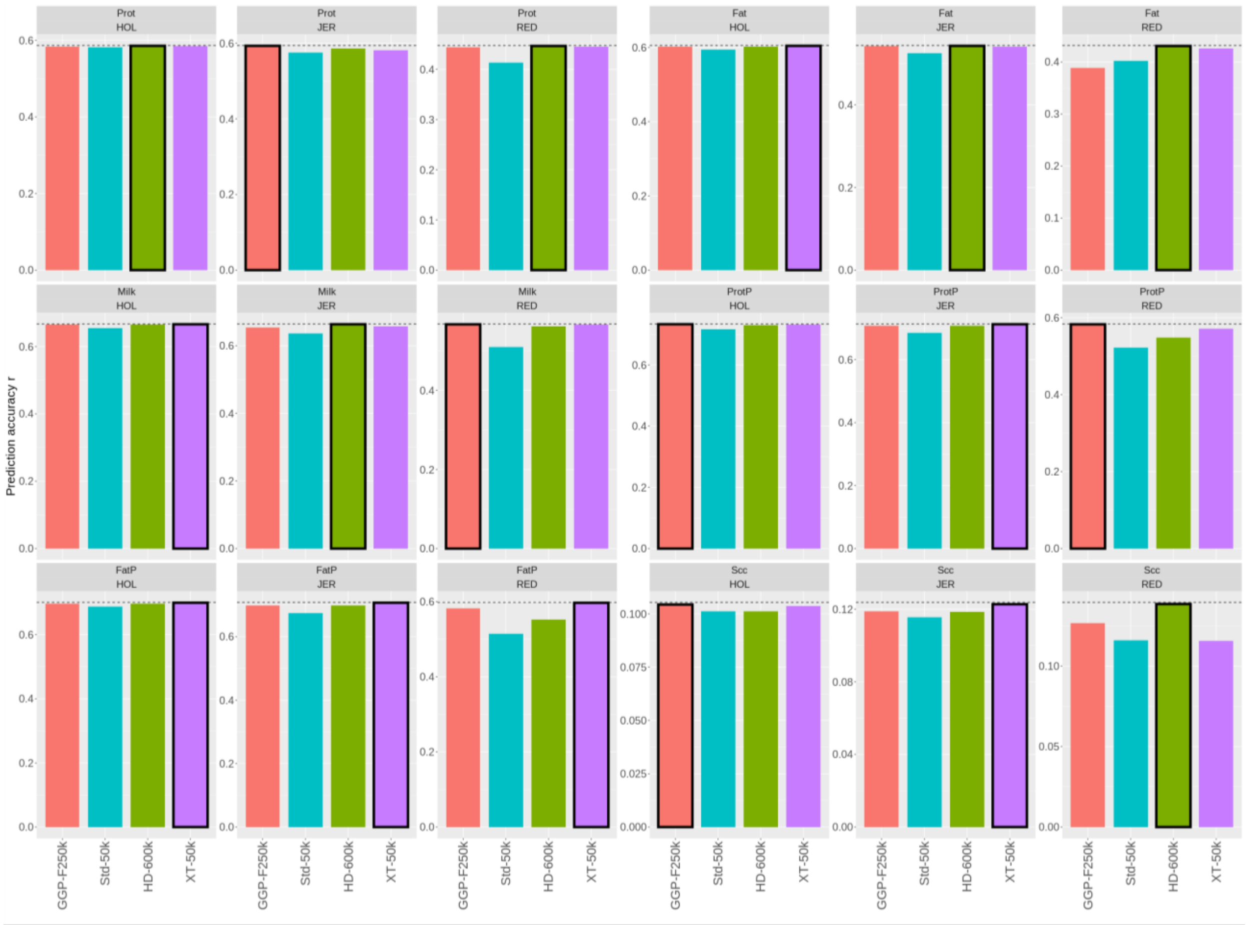

Supplementary Figure 9. Genomic prediction accuracy of variants from 4 different bovine SNP chip panels across 6 traits and 3 breeds. The bar with black border indicates that variant set showing the highest prediction accuracy (*r*) compared to 3 other variant sets in that breed by trait validation. The horizontal black dashed line indicates the prediction accuracy of the best variant set.

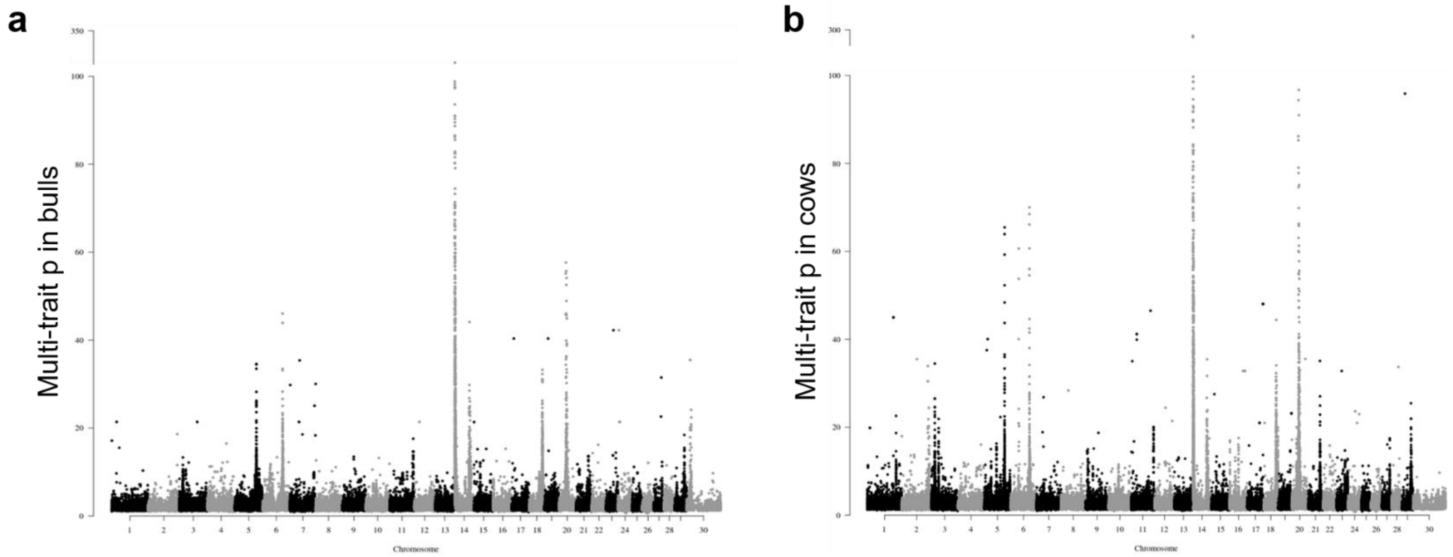

Supplementary Figure 10. Manhattan plots of the 165K variants after prioritization based variant clustering with their multi-trait p values in bulls (A) and cows (B).

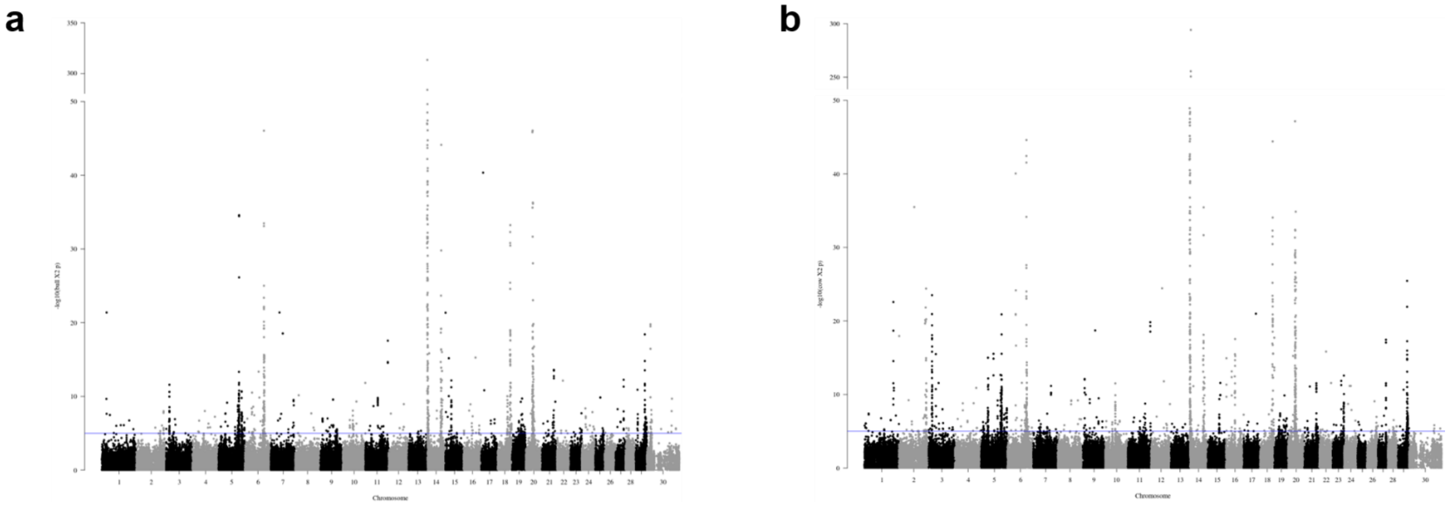

Supplementary Figure 11. Manhattan plots of the 80K variants in bulls (**a**) and cows (**b**), after prioritization based on the cross-sex Bayesian modeling and meta-analysis of local gEBV variance.

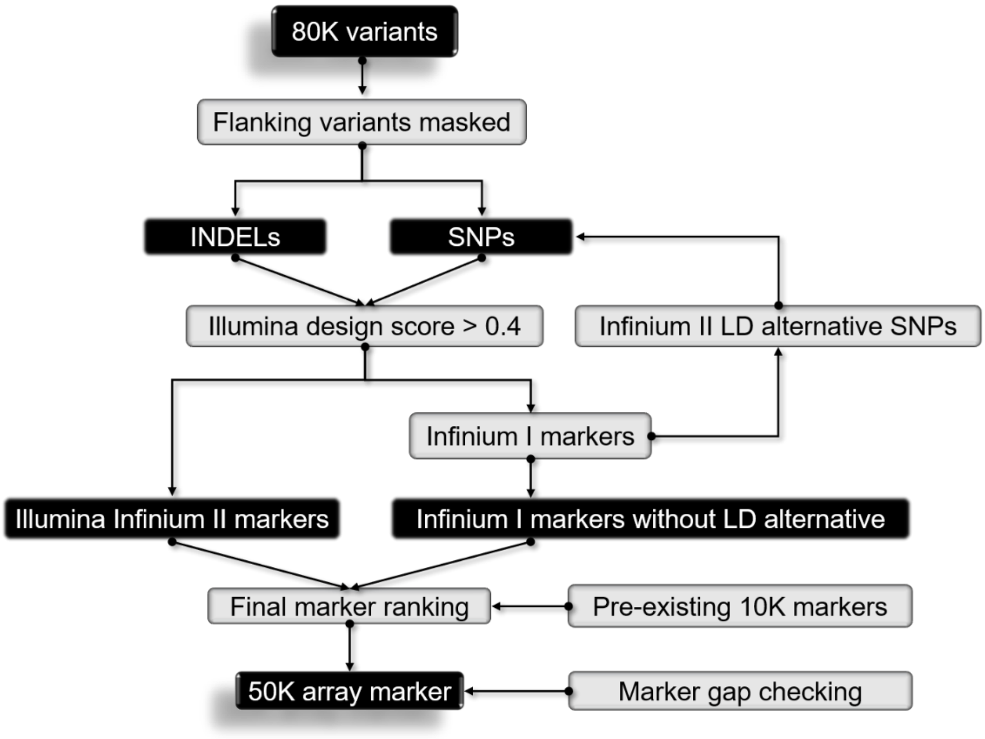

Supplementary Figure 12. The flowchart showing the design process for the 50K-XT array. Flanking sequence for pre-selected 80K variants was extracted and flanking variants masked. DesignStudio (Illumina Inc) was used to calculate design score and designability. Designable markers with a design score > 0.4 were divided into Infinium I markers (occupying 2 beads) and Infinium II markers (occupying 1 bead). For Infinium I markers, iterative searching for LD mates (LD r square > 0.9) from the original variant selection that was Infinium II markers was conducted. All markers were then ranked based on trait-association statistics including the multi-trait p-value and the posterior probability of BayesRC mapping in both sexes. 10K pre-existing markers, including those in previous standard panels and also prioritised by the current analysis, were selected regardless of their bead occupancy and trait-association statistics. Non-pre-existing markers (prioritised by the current study) were then selected in rank order until 50K beads were selected resulting in a total of 46K variants for the new 50K-XT array. The gap between the final marker selection was 57.1±0.4 Kb compared with 65±0.4 Kb for the Standard-50K panel.

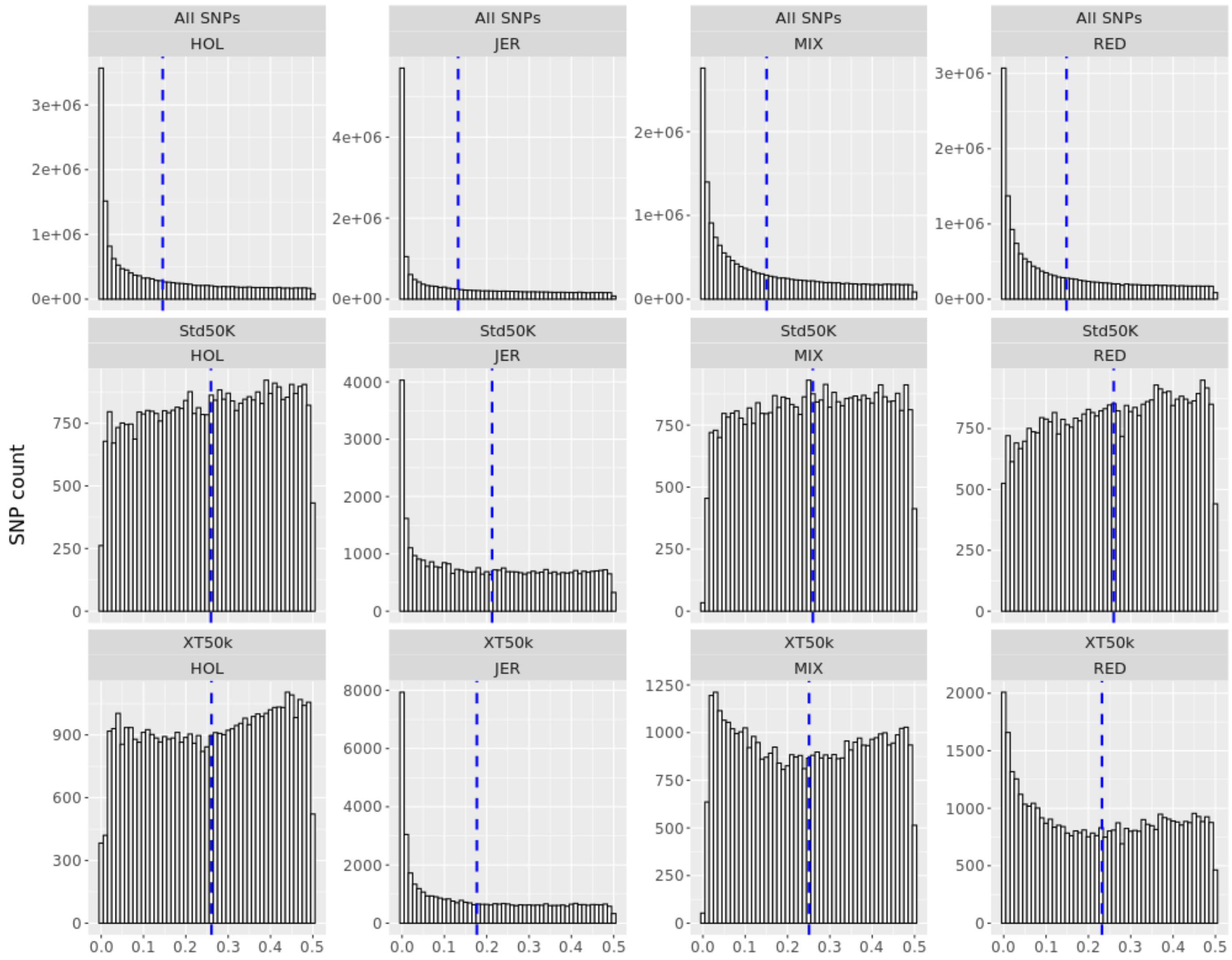

Supplementary Figure 13. Minor allele frequency (MAF) distribution of 17 million variants (all SNPs), variants from the standard-50K panel and from the XT-50K panel prioritised by us in the 44,000 Australian bulls and cows. The blue dashed vertical bars represent the mean of MAF in each panel. HOL: Holstein breed, JER: Jersey breed, MIX: crossbreeds and RED: Australian Red.

Supplementary Table 1. Characteristics of Cholesky decorrelated traits for bulls and cows. The heritability was estimated using all sequence variants with minor allele frequency >0.001.

Trait order	Short name	Full name	Trait type	Number of records in bulls	Trait variance in bulls	Heritability in bulls	Number of records in cows	Trait variance in cows	Heritability of in cows
1	Prot	protein yield	production	11923	1.000	0.7413	32347	1.000	0.388643
2	ProtP	protein percentage	production	11923	0.981	0.8798	32347	1.008	0.675167
3	FatP	fat percentage	production	11923	0.571	0.8127	32347	1.173	0.567957
4	Milk	milk yield	production	11923	1.259	0.8506	29485	0.896	0.693051
5	SCC	somatic cell count	production	11546	1.021	0.7801	26473	0.991	0.270388
6	Fert	fertility	reproduction	11546	0.891	0.5151	26473	1.048	0.0898
7	SurvDi	survival	reproduction	4830	0.872	0.3390	25379	1.024	0.071903
8	Temp	temperament	management	4565	0.963	0.3729	15210	1.011	0.068926
9	MSpeed	milking speed	management	4565	0.944	0.4914	15210	1.017	0.092791
10	Like	likeability	management	4565	0.633	0.2260	15210	1.110	0.041934
11	CentL	central ligament	linear assessment	2908	0.919	0.3925	6658	1.035	0.078289
12	PinW	pin width	linear assessment	2908	0.936	0.4204	6658	1.028	0.181658
13	PinSet	pin set	linear assessment	2908	0.985	0.4928	6658	1.007	0.223049
14	RSet	rear legs set	linear assessment	2908	0.990	0.2383	6658	1.004	0.040869
15	ForeA	fore attachment	linear assessment	2908	0.912	0.2973	6658	1.039	0.103978
16	RearAH	rear attachment height	linear assessment	2908	0.861	0.4609	6658	1.061	0.122225
17	RearAW	rear attachment width	linear assessment	2908	0.895	0.2852	6658	1.046	0.068192
18	TeatPF	front teat placement	linear assessment	2908	0.837	0.5381	6658	1.071	0.219098
19	Stat	stature	linear assessment	2903	0.833	0.5611	6635	1.073	0.200414
20	Angul	angularity	linear assessment	2903	0.925	0.2092	6635	1.033	0.081199
21	Bone	bone quality	linear assessment	2903	0.812	0.3214	6635	1.082	0.119098
22	ChestW	chest width	linear assessment	2903	0.838	0.3209	6635	1.071	0.05169
23	MuzW	muzzle width	linear assessment	2903	0.903	0.3091	6635	1.043	0.104275
24	UdTex	udder texture	linear assessment	2903	0.611	0.0737	6635	1.171	0.016323
25	OType	overall type	linear assessment	2903	0.663	0.1479	6635	1.148	0.099783
26	Mamm	mammary system	linear assessment	2843	0.931	0.3325	6056	1.033	0.106601
27	BodyD	body depth	linear assessment	2660	0.768	0.3602	6051	1.102	0.09744
28	FootA	foot angle	linear assessment	2660	0.883	0.2808	6051	1.052	0.06033
29	TeatL	teat length	linear assessment	2660	0.970	0.5416	6051	1.013	0.216478
30	UdDep	udder depth	linear assessment	2660	0.607	0.3698	6051	1.173	0.096184
31	Loin	loin strength	linear assessment	1880	0.883	0.3752	5901	1.037	0.091612
32	RLeg	rear leg view	linear assessment	1624	0.984	0.2484	5734	1.005	0.074071

33	TeatPR	rear teat placement	linear assessment	1582	0.827	0.4257	5727	1.048	0.078389
34	BCS	body condition score	linear assessment	1439	0.773	0.1364	4086	1.080	0.07249

Supplementary table 2. summary of local gEBV variance (lgebv.var) for 34 traits in each sex.

bull	min	mean	max	cow	min	mean	max
tr01.lgebv.var	0	2.03884E-06	0.014850129	tr01.lgebv.var	0	1.9514E-06	0.008494631
tr02.lgebv.var	0	5.52905E-06	0.011044388	tr02.lgebv.var	0	7.40714E-06	0.019846559
tr03.lgebv.var	0	4.97815E-06	0.06232377	tr03.lgebv.var	0	7.23127E-06	0.048303855
tr04.lgebv.var	0	1.08395E-05	0.026501331	tr04.lgebv.var	0	1.05838E-05	0.016548952
tr05.lgebv.var	0	4.48897E-06	0.004853272	tr05.lgebv.var	0	1.91772E-06	0.019476154
tr06.lgebv.var	0	1.51533E-06	0.00147693	tr06.lgebv.var	0	3.5146E-07	0.002765889
tr07.lgebv.var	0	1.00662E-06	0.00313802	tr07.lgebv.var	0	2.04049E-07	0.00202942
tr08.lgebv.var	0	8.63786E-07	0.005030508	tr08.lgebv.var	0	5.82585E-08	4.20376E-05
tr09.lgebv.var	0	1.08583E-06	0.000564181	tr09.lgebv.var	0	2.56218E-07	0.000383454
tr10.lgebv.var	0	4.16618E-07	0.000255559	tr10.lgebv.var	0	3.80633E-08	0.000337913
tr11.lgebv.var	0	6.8515E-07	0.000973535	tr11.lgebv.var	0	3.3319E-07	8.70659E-05
tr12.lgebv.var	0	9.34312E-07	0.000533084	tr12.lgebv.var	0	8.74747E-07	0.000579623
tr13.lgebv.var	0	1.49131E-06	0.001626246	tr13.lgebv.var	0	1.3518E-06	0.002636798
tr14.lgebv.var	0	7.06329E-07	0.000931157	tr14.lgebv.var	0	3.74399E-07	0.000648484
tr15.lgebv.var	0	7.41879E-07	0.000387428	tr15.lgebv.var	0	5.70505E-07	0.000766782
tr16.lgebv.var	0	8.8107E-07	0.000387732	tr16.lgebv.var	0	6.71761E-07	0.000306892
tr17.lgebv.var	0	6.00062E-07	0.000103444	tr17.lgebv.var	0	4.21623E-07	0.000491854
tr18.lgebv.var	0	1.16572E-06	0.000635738	tr18.lgebv.var	0	9.92409E-07	0.000609962
tr19.lgebv.var	0	1.64528E-06	0.00155134	tr19.lgebv.var	0	1.65588E-06	0.004215092
tr20.lgebv.var	0	1.09575E-06	0.000139662	tr20.lgebv.var	0	5.08806E-07	0.001071604
tr21.lgebv.var	0	1.03857E-06	0.000180351	tr21.lgebv.var	0	1.88637E-06	0.004557177
tr22.lgebv.var	0	5.91384E-07	0.000464256	tr22.lgebv.var	0	4.43038E-07	0.001395866
tr23.lgebv.var	0	5.52088E-07	0.001265194	tr23.lgebv.var	0	5.31408E-07	0.000361651
tr24.lgebv.var	0	1.753E-07	2.1599E-05	tr24.lgebv.var	0	2.23492E-07	3.10429E-05
tr25.lgebv.var	0	2.95555E-07	0.000103169	tr25.lgebv.var	0	2.97965E-07	0.000380383
tr26.lgebv.var	0	5.99923E-07	0.004330949	tr26.lgebv.var	0	3.33054E-07	0.000244207
tr27.lgebv.var	0	6.5653E-07	0.000336248	tr27.lgebv.var	0	5.18959E-07	0.00021204
tr28.lgebv.var	0	7.38505E-07	0.001607027	tr28.lgebv.var	0	2.99968E-07	0.000208941
tr29.lgebv.var	0	1.80189E-06	0.003947483	tr29.lgebv.var	0	9.73451E-07	0.000472375
tr30.lgebv.var	0	5.08624E-07	0.000126041	tr30.lgebv.var	0	5.15868E-07	6.15572E-05
tr31.lgebv.var	0	1.14246E-06	0.00079715	tr31.lgebv.var	0	3.27328E-07	0.000135469
tr32.lgebv.var	0	7.64049E-07	0.000130779	tr32.lgebv.var	0	3.22681E-07	0.000152997
tr33.lgebv.var	0	1.2378E-06	0.001249247	tr33.lgebv.var	0	5.22815E-07	7.30413E-05
tr34.lgebv.var	0	3.2396E-07	7.22906E-05	tr34.lgebv.var	0	3.90159E-07	0.000169495

Supplementary Table 3. Overlap of traits between Australian (AUS) and US data.

AUS trait order	AUS trait full name	AUS trait short name	equivalent US trait short name	equivalent US trait full name	short name of US trait not in AUS data	full name of US trait not in AUS data
1	protein yield	Prot	Protein	Protein yield	Net_Merit	Net merit
2	protein percentage	ProtP	Pro_Percent	Protein percentage	AFC	Age at first calving
3	fat percentage	FatP	Fat_Percent	Fat percentage	DFB	Days to firrst breedinga
4	milk yield	Milk	Milk	Milk yield	Heifer_Conc_Rate	Heifer conception rate
5	somatic cell count	SCC	SCS	Somatic cell score	Cow_Conc_Rate	Cow conception rate
6	fertility	Fert	Dtr_Preg_Rate	Daughter pregnancy rate	Sire_Calv_Ease	Sire calving ease
7	survival	SurvDi	Prod_Life	Productive life	Dtr_Calv_Ease	Daughter calving ease
8	temperament	Temp			Sire_Still_Birth	Sire stillbirth
9	milking speed	MSpeed			Dtr_Still_Birth	Daughter stillbirth
10	likeability	Like			Final_score	Final score
11	central ligament	CentL			Strength	Strength
12	pin width	PinW	Rump_width	Rump width	Dairy_form	Dairy form
13	pin set	PinSet	Rump_angle	Rump angle	Rear_legs(side)	Rear legs (side view)
14	rear legs set	RSet			Rear_ud_height	Rear udder height
15	fore attachment	ForeA	Fore_udder_att	Fore udder attachment	Udder_cleft	Udder cleft
16	rear attachment height	RearAH			Feet_and_legs	Feet and legs composite
17	rear attachment width	RearAW			CFI	Days from calving to first insemination
18	front teat placement	TeatPF	Front_teat_pla	Front teat placement		
19	stature	Stat	Stature	Stature		
20	angularity	Angul				
21	bone quality	Bone				
22	chest width	ChestW				
23	muzzle width	MuzW				
24	udder texture	UdTex				
25	overall type	OType				
26	mammary system	Mamm				
27	body depth	BodyD	Body_depth	Body depth		
28	foot angle	FootA	Foot_angle	Foot angle		
29	teat length	TeatL	Teat_length	Teat length		
30	udder depth	UdDep	Udder_depth	Udder depth		
31	loin strength	Loin				

32	rear leg view	RLeg	Rear_legs(rear)	Rear legs (rear view)
33	rear teat placement	TeatPR	Rear_teat_pla	Rear teat placement
34	body condition score	BCS		

US trait details can be found in: Jiang J, et al. (2019) Functional annotation and Bayesian fine-mapping reveals candidate genes for important agronomic traits in Holstein bulls. Communications Biology 2(1):212.

Supplementary References:

1. MacLeod I, Bowman P, Vander Jagt C, Haile-Mariam M, Kemper K, Chamberlain A, et al. Exploiting biological priors and sequence variants enhances QTL discovery and genomic prediction of complex traits. BMC genomics. 2016;17(1):144.
2. Erbe M, Hayes B, Matukumalli L, Goswami S, Bowman P, Reich C, et al. Improving accuracy of genomic predictions within and between dairy cattle breeds with imputed high-density single nucleotide polymorphism panels. Journal of dairy science. 2012;95(7):4114-29.
3. Yang J, Lee SH, Goddard ME, Visscher PM. GCTA: a tool for genome-wide complex trait analysis. The American Journal of Human Genetics. 2011;88(1):76-82.
4. Xiang R, Berg Ivd, MacLeod IM, Hayes BJ, Prowse-Wilkins CP, Wang M, et al. Quantifying the contribution of sequence variants with regulatory and evolutionary significance to 34 bovine complex traits. Proceedings of the National Academy of Sciences. 2019;116(39):19398-408. doi: 10.1073/pnas.1904159116.
5. Xiang R, van den Berg I, MacLeod IM, Daetwyler HD, Goddard ME. Effect direction meta-analysis of GWAS identifies extreme, prevalent and shared pleiotropy in a large mammal. Commun Biol. 2020;3(1):88. Epub 2020/03/01. doi: 10.1038/s42003-020-0823-6. PubMed PMID: 32111961; PubMed Central PMCID: PMCPMC7048789.
6. Rowan TN, Hoff JL, Crum TE, Taylor JF, Schnabel RD, Decker JE. A multi-breed reference panel and additional rare variants maximize imputation accuracy in cattle. Genetics Selection Evolution. 2019;51(1):1-16.
7. McLaren W, Gil L, Hunt SE, Riat HS, Ritchie GRS, Thormann A, et al. The Ensembl Variant Effect Predictor. Genome Biology. 2016;17(1):122. doi: 10.1186/s13059-016-0974-4.
8. Kemper KE, Reich CM, Bowman PJ, Vander Jagt CJ, Chamberlain AJ, Mason BA, et al. Improved precision of QTL mapping using a nonlinear Bayesian method in a multi-breed population leads to greater accuracy of across-breed genomic predictions. Genetics Selection Evolution. 2015;47(1):29.
