# Supplementary material for: Genome-wide fine-mapping identifies pleiotropic and functional variants that predict many traits across global cattle populations
Source: Nat Commun. 2021 Feb 8;12:860. doi: 10.1038/s41467-021-21001-0 (PMC7870883; doi:10.1038/s41467-021-21001-0)
Supplement: Supplementary file 3 — Reporting Summary [file 41467_2021_21001_MOESM3_ESM.pdf]

nature research

Corresponding author(s): Ruidong Xiang

Last updated by author(s): Jan 6, 2021

Reporting Summary

Nature Research wishes to improve the reproducibility of the work that we publish. This form provides structure for consistency and transparency in reporting. For further information on Nature Research policies, see our Editorial Policies and the Editorial Policy Checklist.

Statistics

For all statistical analyses, confirm that the following items are present in the figure legend, table legend, main text, or Methods section.

n/a	Confirmed	
☐	☒	The exact sample size (*n*) for each experimental group/condition, given as a discrete number and unit of measurement
☐	☒	A statement on whether measurements were taken from distinct samples or whether the same sample was measured repeatedly
☐	☒	The statistical test(s) used AND whether they are one- or two-sided<br>*Only common tests should be described solely by name; describe more complex techniques in the Methods section.*
☐	☒	A description of all covariates tested
☐	☒	A description of any assumptions or corrections, such as tests of normality and adjustment for multiple comparisons
☐	☒	A full description of the statistical parameters including central tendency (e.g. means) or other basic estimates (e.g. regression coefficient) AND variation (e.g. standard deviation) or associated estimates of uncertainty (e.g. confidence intervals)
☐	☒	For null hypothesis testing, the test statistic (e.g. *F*, *t*, *r*) with confidence intervals, effect sizes, degrees of freedom and *P* value noted<br>*Give P values as exact values whenever suitable.*
☐	☒	For Bayesian analysis, information on the choice of priors and Markov chain Monte Carlo settings
☐	☒	For hierarchical and complex designs, identification of the appropriate level for tests and full reporting of outcomes
☐	☒	Estimates of effect sizes (e.g. Cohen's *d*, Pearson's *r*), indicating how they were calculated

Our web collection on statistics for biologists contains articles on many of the points above.

Software and code

Policy information about availability of computer code

Data collection	no software was used for data collection.
Data analysis	The GWAS used plink (https://www.cog-genomics.org/plink/) and GCTA (https://cnsgenomics.com/software/gcta/#Overview). The multi-trait meta-analysis implemented in R is published at https://melbourne.figshare.com/articles/Effect_Direction_MEta-analysis_EDME_of_GWAS/11730939/1. Variant annotation used public Variant Effect Prediction of Ensembl and NGS-SNP http://stothard.afns.ualberta.ca/downloads/NGS-SNP/. The details of BayesRC can be found at https://bmcgenomics.biomedcentral.com/articles/10.1186/s12864-016-2443-6. The implementation of weighted correlation analysis is detailed in Supplementary Note S2. The R codes of variant clustering with test datasets are available at https://github.com/rxiangr/SNP_cluster_ranking and the R codes of correlation between genotypes and local gEBV with its test datasets are available at https://github.com/rxiangr/SNP_correlation_local_gEBV_variance.

For manuscripts utilizing custom algorithms or software that are central to the research but not yet described in published literature, software must be made available to editors and reviewers. We strongly encourage code deposition in a community repository (e.g. GitHub). See the Nature Research guidelines for submitting code & software for further information.

Data

Policy information about availability of data

All manuscripts must include a data availability statement. This statement should provide the following information, where applicable:

- Accession codes, unique identifiers, or web links for publicly available datasets
- A list of figures that have associated raw data
- A description of any restrictions on data availability

The information regarding the XT-50K markers and the multi-trait GWAS summary statistics of the 46.5k markers of Australian bulls, cows and the US bulls are accessible via figshare at https://figshare.com/s/93bd992a42786f9466b7 with the DOI: 10.26188/13523837. The functional and evolutionary data are public

available via the FAETH score at “https://melbourne.figshare.com/articles/The_Functional_And_Evolutionary_Trait_Heritability_FAETH_score_of_over_17_million_cattle_sequence_variants/7660277/2”. Additional GWAS results for Australian animals can be found at Xiang et al 2020 24. DataGene Australia (http://www.datagene.com.au/) are custodians of the raw phenotype and genotype data of Australian farm animals. DairyNZ (https://www.dairynz.co.nz/) are custodians of the raw phenotype data of New Zealand farm animals and CRV (https://www.crv4all-international.com/) are custodians of the raw genotypes of New Zealand farm animals. Details of the data access to the GWAS results from US animals used for validation of XT-50K markers can be found at Jiang et al 2019 29. The DNA sequence data as part of the 1000 bull genome project 4,34,48 is included in NCBI BioProjects PRJNA431934 (https://www.ncbi.nlm.nih.gov/bioproject/PRJNA431934/), PRJNA238491 (https://www.ncbi.nlm.nih.gov/bioproject/?term=PRJNA238491), PRJDB2660 (https://www.ncbi.nlm.nih.gov/bioproject/?term=PRJDB2660), PRJEB18113 (https://www.ncbi.nlm.nih.gov/bioproject/356238), PRJEB1829 (https://www.ncbi.nlm.nih.gov/bioproject/?term=PRJEB1829), PRJEB27309 (https://www.ncbi.nlm.nih.gov/bioproject/?term=PRJEB27309), PRJEB28191 (https://www.ncbi.nlm.nih.gov/bioproject/?term=PRJEB28191), PRJEB9343 (https://www.ncbi.nlm.nih.gov/bioproject/292988), PRJNA210519 (https://www.ncbi.nlm.nih.gov/bioproject/?term=PRJNA210519), PRJNA210521 (https://www.ncbi.nlm.nih.gov/bioproject/?term=PRJNA210521), PRJNA210523 (https://www.ncbi.nlm.nih.gov/bioproject/?term=PRJNA210523), PRJNA279385 (https://www.ncbi.nlm.nih.gov/bioproject/?term=PRJNA279385), PRJNA294709, PRJNA316122 (https://www.ncbi.nlm.nih.gov/bioproject/?term=PRJNA316122), PRJNA474946 (https://www.ncbi.nlm.nih.gov/bioproject/?term=PRJNA474946), PRJNA477833 (https://www.ncbi.nlm.nih.gov/bioproject/?term=PRJNA477833), PRJNA494431 (https://www.ncbi.nlm.nih.gov/bioproject/?term=PRJNA494431), PRJDA48395 (https://www.ncbi.nlm.nih.gov/bioproject/?term=PRJDA48395), PRJNA431934 (https://www.ncbi.nlm.nih.gov/bioproject/?term=PRJNA431934), PRJNA238491 (https://www.ncbi.nlm.nih.gov/bioproject/?term=PRJNA238491). Other supporting data are shown in the supplementary materials of the current manuscript.

Field-specific reporting

Please select the one below that is the best fit for your research. If you are not sure, read the appropriate sections before making your selection.

☒ Life sciences ☐ Behavioural & social sciences ☐ Ecological, evolutionary & environmental sciences

For a reference copy of the document with all sections, see nature.com/documents/nr-reporting-summary-flat.pdf

Life sciences study design

All studies must disclose on these points even when the disclosure is negative.

Sample size	We used all available data which has a discovery sample size > 44,000 cattle.
Data exclusions	All data are included.
Replication	We used validation datasets > 90,000 cattle from the USA, Australia and New Zealand.
Randomization	No groups are introduced.
Blinding	There was no groups introduced, therefore, the blinding is irrelevant to the study.

Reporting for specific materials, systems and methods

We require information from authors about some types of materials, experimental systems and methods used in many studies. Here, indicate whether each material, system or method listed is relevant to your study. If you are not sure if a list item applies to your research, read the appropriate section before selecting a response.

Materials & experimental systems

n/a	Involved in the study	
☒	☐	Antibodies
☒	☐	Eukaryotic cell lines
☒	☐	Palaeontology and archaeology
☒	☐	Animals and other organisms
☒	☐	Human research participants
☒	☐	Clinical data
☒	☐	Dual use research of concern

Methods

n/a	Involved in the study	
☒	☐	ChIP-seq
☒	☐	Flow cytometry
☒	☐	MRI-based neuroimaging
